# Rapid and sensitive determination of Se and heavy metals in foods using electrothermal vaporization inductively coupled plasma mass spectrometry with a novel transportation system

**DOI:** 10.3389/fnut.2023.1201801

**Published:** 2023-06-07

**Authors:** Guanyu Lan, Xue Li, Jijun Yao, Xiaofeng Yu, Qinghai Liu, Cheng Qiu, Xuefei Mao

**Affiliations:** ^1^Institute of Agricultural Product Quality Standard and Testing Research, Tibet Academy of Agricultural and Animal Husbandry Sciences, Lhasa, Tibet, China; ^2^Institute of Quality Standard and Testing Technology for Agro-Products, Key Laboratory of Agro-food Safety and Quality, Ministry of Agriculture and Rural Affairs, Chinese Academy of Agricultural Sciences, Beijing, China; ^3^Hangzhou Puyu Technology Co. Ltd., Hangzhou, Zhejiang, China

**Keywords:** selenium, heavy metals, electrothermal vaporization, slurry sampling, signal delay device, inductively coupled plasma mass spectrometry

## Abstract

Rapid, sensitive and simultaneous determination of trace multi-elements in various plant food samples such as grain, oilseed, vegetable and tea is always a challenge thus far. In this work, a rapid determination method for Se, Cd, As and Pb in food samples by inductively coupled plasma mass spectrometry (ICP-MS) using slurry sampling electrothermal vaporization (SS-ETV) was developed. To improve the analytical sensitivity and precision as well as eliminate the memory effect, a gas turbulator line and signal delay device (SDD) were for the first time designed for the graphite furnace (GF) ETV coupled with ICP-MS. The signal acquisition parameters of ICP-MS, ashing and vaporization conditions, and the flow rates of carrier gas and gas turbulator were investigated for Se, Cd, As and Pb in food samples. Under the optimized conditions, the limits of determination (LODs) for Se, Cd, As and Pb were 0.5 ng g^−1^, 0.3 ng g^−1^, 0.3 ng g^−1^ and 0.6 ng g^−1^, respectively; the limits of quantification (LOQs) for Se, Cd, As and Pb were 1.7 ng g^−1^, 1.0 ng g^−1^, 1.0 ng g^−1^ and 1.9 ng g^−1^, respectively; linearity (*R^2^*) in the range of 1 to 4,000 ng g^−1^ was >0.999 using the standard addition method. This method was used to analyze 5 CRMs including rice, tea and soybeans, and the concentrations detected by this method were within the range of the certified values. The recoveries of Se, Cd, As and Pb in plant food matrices including grain, oilseed, celery, spinach, carrot and tea samples were 86–118% compared to the microwave digestion ICP-MS method; and the relative standard deviations (RSDs) were 1.2–8.9% for real food sample analysis, proving a good precision and accuracy for the simultaneous determination of multi-elements. The analysis time was less than 3 min, slurry preparation time < 5 min without sample digestion process. The proposed direct slurry sampling ICP-MS method is thus suitable for rapid and sensitive determination of Se, Cd, As and Pb in food samples with advantages such as simplicity, green and safety, as well as with a promising application potential in detecting more elements to protect food safety and human health.

## 1. Introduction

Selenium-enriched food is increasingly becoming a popular research focus in recent years, considering Se (Selenium) is an indispensable component for more than 30 kinds of selenoproteins and selenium containing enzymes, playing important roles in anti-oxidation, anti-cancer activity, and improvement of human immunity (1). However, the human body does not synthesize selenium by itself and needs to intake selenium via foods, drinking, or supplementary food (2). However, selenium is often linked to other metal sulfide deposits and rock coal, resulting in the coexistence of heavy metals in the naturally selenium-rich soils (3). Further, agri-food with selenium is also enriched with co-existing heavy metals (4). As well, a study reported a high correlation between heavy metal content in soil with the selenium content (5). The co-existing heavy metals such as cadmium, arsenic, lead and so on are a potential risk to human health. Acute poisoning by arsenic can lead to acute gastroenteritis and polyneuritis (6); cadmium causes dysregulation of calcium metabolism, renal tubular dysfunction, neurotoxicity, and osteoporosis (7); lead has a wide range of negative effects on the human nervous system, cardiovascular system, and skeletal system (8). Thus, fast and accurate determination of Cd (Cadmium), As (Arsenic), and Pb (Lead) in selenium-enriched food is crucial for food safety and human health.

At present, the frequently used analysis methods for heavy metals are graphite furnace/flame atomic absorption (GF/F-AAS), hydride generation atomic fluorescence spectrometry (HG-AFS), inductively coupled plasma mass spectrometry (ICP-MS) and inductively coupled plasma optical emission spectrometry (ICP-OES) with liquid sampling system (9–13). The instrumental approaches mentioned above require a complicated sample digestion process with the use of strong acids or alkali reagents. This is indeed time consuming and environmental unfriendly, leading to an impossibility of green determination of Se, Cd, As, and Pb in food samples.

In contrast to pneumatic atomization, common direct sampling methods include electrothermal vaporization (ETV), laser ablation (LA) and laser induced breakdown spectroscopy (LIBS). ETV is one of sample introduction allowing the direct sampling of solid or slurry with a high sampling efficiency and a small sample size (14). The ETV materials include graphite, ceramics, and high melting point metals to be fabricated as tube, boat, coils, and foil shapes (15, 16) to couple with various atomic spectrometers such as ICP-MS/OES, AAS, and AFS. Among them, ICP-MS demonstrates a multi-elemental analysis capacity with the highest sensitivity and dynamic linearity range. Li et al. applied ultrasonic slurry sampling for the determination of multi-elements in powdered rice sample using a commercial electrothermal vaporizer coupled with ICP-MS, in which NH_3_ was used as a reaction gas to reduce the background signal of Cr, and ascorbic acid to enhance the ion signal. The limits of detection (LODs) of Cr, Cu, Cd, Hg, and Pb reached 0.4–1.7 ng g^−1^ (17). However, considering the elemental introduction by ETV is a rapid and transient process for determination, the short signal monitoring window results in insufficient signal acquisition by ICP-MS thereby affecting the analysis precision (18). If the time resolution cannot be improved significantly, the solid sampling ETV device should be modified to solve this problem.

Further, it must be noticed that mass spectrometric interference is an important impact factor for analysis precision and accuracy including double charge interferences of rare earth elements (REEs), oxide formation, and matrix containing primary elements. For example, ^59^Co^16^O interferes with ^75^As, ^16^O^1^H^65^Cu interferes with ^82^Se, ^95^Mo^16^O interferes with ^111^Cd, ^192^Os^16^O interferes with ^208^Pb, and so on. The multi-response mode of ICP-MS/MS is very effective in eliminating interferences (19), but it does not work as well for ETV, mainly because of the transient signal acquisition during the ETV. ETV heating process enables the removal of most matrices via sample dehydration and ashing; and partial interference can be avoided via gradient temperature rise due to the vaporization temperature difference of target elements. However, accurate and precise multi-elemental analysis is still a challenge for complicated food matrix containing fat, protein and starch. Herein, elemental compounds are variable existing in food matrix and require various vaporization temperature, possibly resulting in split signal peaks and affecting analytical precision. Meanwhile, the “carry-on effect” and “residual effect” induced by microparticles may also impact the transport efficiency and even the ionization efficiency of aerosols. To eliminate matrix interference, chemical modifiers includ Pd/Mg salts (20), TritonX-100 (21), ascorbic acid (22), and fluorinated agents (23) are often used in ETV applications. However, most chemical modifiers have a very limited use when sampling directly on solids, considering the difficulties of adequate mixing and contact (24). This problem is well resolved when using slurry sampling, as it has the additional advantages of being easily calibrated (matrix matching or standard addition) and automated (25).

To accomplish the simultaneous determination of Se, Cd, As and Pb in various food samples including grain, oilseed, vegetable and tea, a graphite furnace-based ETV-ICP-MS for slurry sampling analysis was developed. Herein, an external signal delay device (SDD) with the matched gas turbulator line system was for the first time designed and applied to ETV-ICP-MS to improve the analysis precision and sensitivity. Also, the atmosphere system, thermal control system and series interface of the ETV unit were optimized to accommodate the requirements of using various food samples. Under optimized conditions, the LODs of Se, Cd, As and Pb reached 0.5 ng g^−1^, 0.3 ng g^−1^, 0.3 ng g^−1^ and 0.6 ng g^−1^, respectively; The LOQs for Se, Cd, As and Pb were 1.7 ng g^−1^, 1.0 ng g^−1^, 1.0 ng g^−1^ and 1.9 ng g^−1^, respectively. The proposed method demonstrated advantage such as rapidness, efficiency, and accuracy for the simultaneous multi-elemental determination in food samples.

## 2. Materials and methods

### 2.1. Instrumentation

**Figure 1:**
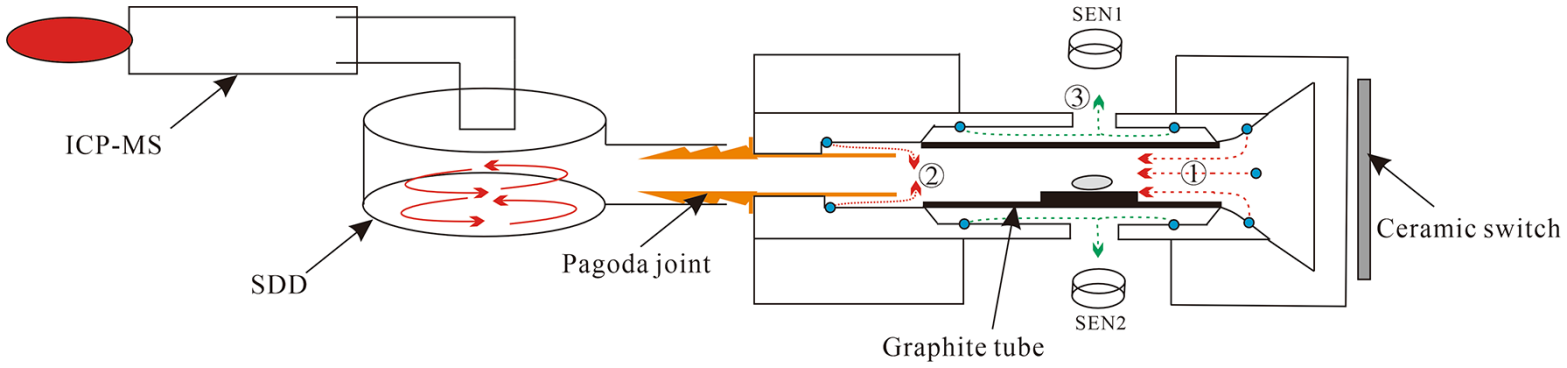
Schematic diagram of ETV-ICP-MS instrumentation. SDD refers to a signal delay device. SEN1 and SEN2 refer to a photoelectric sensor and an infrared sensor, respectively. ①②③ refer to the carrier gas line, gas turbulator line, and protection gas line, respectively.

The direct slurry sampling electrothermal vaporization (DSS-ETV) system was fabricated in our laboratory, and its schematic diagram is shown in Figure 1. The DSS-ETV mainly consists of a graphite furnace as a vaporizer, a temperature sensor and control system, a cooling system, a gas line system, an interface with ICP-MS and a power supply. Graphite tube (28 × 7.4 mm, ɸ5.9 mm, Beijing Xiangchenghe Photoelectric Technology Co., Ltd., Beijing, China) was employed as a sampling boat in the graphite furnace to heat (longitudinally with graphite electrodes) a slurry sample; as well, it was coated with pyrolytic carbon via heating in methane and ammonia according to the previous study (26). To monitor the heating temperature of ETV, photoelectric sensors (S1226-44BQ, Beijing Hamamatsu Photoelectric Technology Co., Ltd., Beijing, China) were used for room temperature to 1,200°C, and infrared sensors (SA120BSK, Wuxi Shiao Technology Co., Wuxi, China) for 1,000 to 2,600°C. The ETV temperature from room temperature to 2,600°C was controlled by a proportional integral derivative (PID) algorithm based on a computer and power supply (AC, 50 Hz). The cooling system consists of water and air cooling lines, in which cooling water was fed into the copper electrode holders and cooling gas flowed between the graphite tube and the graphite furnace. The gas line system was controlled by two gas mass flow meters (GMFC) and a manual valve for carrier gas, gas turbulator and protection gases, respectively. The ETV was connected to an ICP-MS (SUPEC 7000, Hangzhou Puyu Technology Development Co., Ltd., Hangzhou, China) by a cyclone nebulizer (internal capacity ~40 mL) made of polyvinylidene fluoride (PFA), which acts as an external signal delay device to improve the analysis precision. The detailed parameters of the ETV-ICP-MS instrumentation are shown in Supplementary Tables S1, S2.

To validate the proposed method, ICP-MS was used for the determination of Se, Cd, As and Pb in food samples after microwave digestion. HNO_3_ and H_2_O_2_ (guaranteed grade) were purchased from Sinopharm Chemical Regent Co., Ltd. (Shanghai, China). The detailed operating conditions of the microwave digestion system (790S, Hangzhou Puyu Technology Development Co., Ltd., Hangzhou, China) and ICP-MS (SUPEC 7000, Hangzhou Puyu Technology Development Co., Ltd., Hangzhou, China) are shown in Supplementary Table S3.

### 2.2. Chemicals

Standard solutions (1,000 mg L^−1^) of Se, Cd, As and Pb were purchased from the National Center for Analysis and Testing of Nonferrous Metals and Electronic Materials (Shanghai, China). Deionized (DI) water (15 MΩ) was prepared by a Milli-Q Elix Essential purification system (Millipore, Burlington, MA, United States). Working standards were obtained by diluting the stock solutions. The certified reference materials (CRMs) of rice (GBW10010a and GBW10045a), soybean (GBW10013), and tea (GBW10016a and GBW10052a) were purchased from the National Research Center for Reference Materials (Beijing, China). HNO_3_ and H_2_O_2_ (guaranteed grade) were used for sample digestion.

### 2.3. Analytical procedures of direct slurry sampling ETV-ICP-MS

The analytical procedures for the determination of Se, Cd, As and Pb in food samples by the proposed ETV-ICP-MS method are described as follows: (1) Dehydration: 10 μL of slurry sample was taken into the graphite tube and kept at 200°C for 15 s to remove moisture in sample, (2) Ashing: ETV was heated to 450°C within 20 s and held for 20 s under 100 mL min^−1^ carrier gas and 300 mL min^−1^ gas turbulator, respectively, to remove organic substances in sample outside via an outlet, (3) Vaporization: After shutting down the outlet, the carrier and gas turbulatores were adjusted to 500 mL min^−1^ and 600 mL min^−1^, respectively and kept for 5 s to form a stable working atmosphere. Then, the ETV was heated to 2,300°C within 1 s and then held for 5 s to vaporize Se, Cd, As and Pb analytes, (4) Detection: The vaporized analytes were transported into the SDD and then mixed into the ICP source for the quantitative analysis by the quadrupole mass spectrometer, (5) Cleaning: 2500°C for 7 s was performed under 500 mL min^−1^ carrier gas and 800 mL min^−1^ gas turbulator to remove possible residues in the instrumental system.

### 2.4. Microwave digestion ICP-MS method

According to the Chinese national standard of GB 5009.268 (27), food samples were measured for the presences of Se, Cd, As and Pb to verify the proposed method. A 1 g food sample was weighed and then mixed with 1 mL DI water, 2 mL HNO_3_ and 1 mL H_2_O_2_ in modified tetrafluoroethylene (TFM) digestion tube. After the microwave digestion as shown in Supplementary Table S3, the sample solution was cooled to room temperature in the end. The final sample solution was transferred to a 50 mL centrifuge tube and diluted to 15 mL with 2% HNO_3_ (v:v). The digested solution was detected by ICP-MS using He collision cell mode as shown in Supplementary Table S4.

### 2.5. Slurry sample preparation

Celery, spinach, and carrot samples were purchased from local supermarkets (Hangzhou, China), and then washed and aired at ambient temperature; subsequently, cut into pieces and dehydrated for 48 h at ~50°C in an oven (101-3B, Shanghai Lianjing Electronic Technology Co., Ltd., Shanghai, China). The dried samples were ground into powder by a grinder (BJ-800A, Deqing Baijie Electric Co., Ltd., Huzhou, China) and then sieved through 60 mesh. According to the previous studies (28, 29), 0.05 g celery, spinach, carrot or tea sample was placed in a crushing tube with 8 zirconium balls (ɸ2.0 mm) and mixed with 1 mL of 0.5% (v:v) Triton X-100 (PerkinElmer, Waltham, MA, United States) solution; for grain sample, ɸ1.5 mm zirconium balls were employed. After sealing, the crushing tube was put into a grinder (DS-100, Hubei Xinzongke Virus Engineering Technology Co., Ltd., Wuhan, China) and shaken for 3 times with 15 s each time at 5500 rpm. After grinding, the sample will be shaken again for 1 min using a vortex mixer, and measured by the proposed method as soon as possible. The prepared slurry does not need to be separated from the zirconium beads and can be sampled directly using a pipette gun. The slurry sample preparation time was controlled within 5 min, and grinder can process dozens of samples at one time in 1 min, enabling high throughput sample treatment.

### 2.6. Statistical analysis

Statistical analysis of experimental data was performed using Origin 2021 and Microsoft^R^ Excel 2019. Significant differences were assessed by t-test, in which *p*-values lower than 0.05 (*p* < 0.05) support statistical significance, t-test is two-sided. The “green” performance of the method was evaluated using the software “AGREE.”

## 3. Results

### 3.1. Design of solid sampling ETV-ICP-MS instrumentation

The graphite furnace was first used as an ETV by L’vov in the 1950s in coupling with AAS. Nowadays, graphite furnaces are also the most common devices for ETV-ICP-MS. However, the commercialized graphite furnace for AAS is not professionally designed for ICP-MS, so the hyphenated gas line system and analyte transportation must be modified and improved for ICP-MS.

#### 3.1.1. Design of gas line system

In the conventional graphite furnace, carrier gas was designed to carry the elemental analyte. So, at first, the graphite furnace ETV with only carrier gas line was employed to vaporize food sample, however the deposition of matrix residue was obviously found on the outlet of the graphite furnace (Supplementary Figure S1) after 50 repeated introductions. Herein, resistance and absorption of the pagoda joint and lower temperature vs. the heating area in the furnace makes vaporized aerosols more readily condensed on the inner surface of the outlet (30). To deal with the deposition of analyte transported, a new gas turbulator line of annular and symmetrical tetra-holes in the downstream graphite furnace (Supplementary Figure S2) was designed for the ETV device. The supplemented gas turbulator mixed with carrier gas to form a turbulence to alleviate the directed deposition, as well as accelerate analyte aerosol to pass the outlet of the pagoda joint. The picture in Supplementary Figure S1 demonstrates a clean pagoda joint after 50 repeated introductions, proving an effective improvement of analyte transportation to avoid memory effect for graphite furnace ETV.

**Figure 2:**
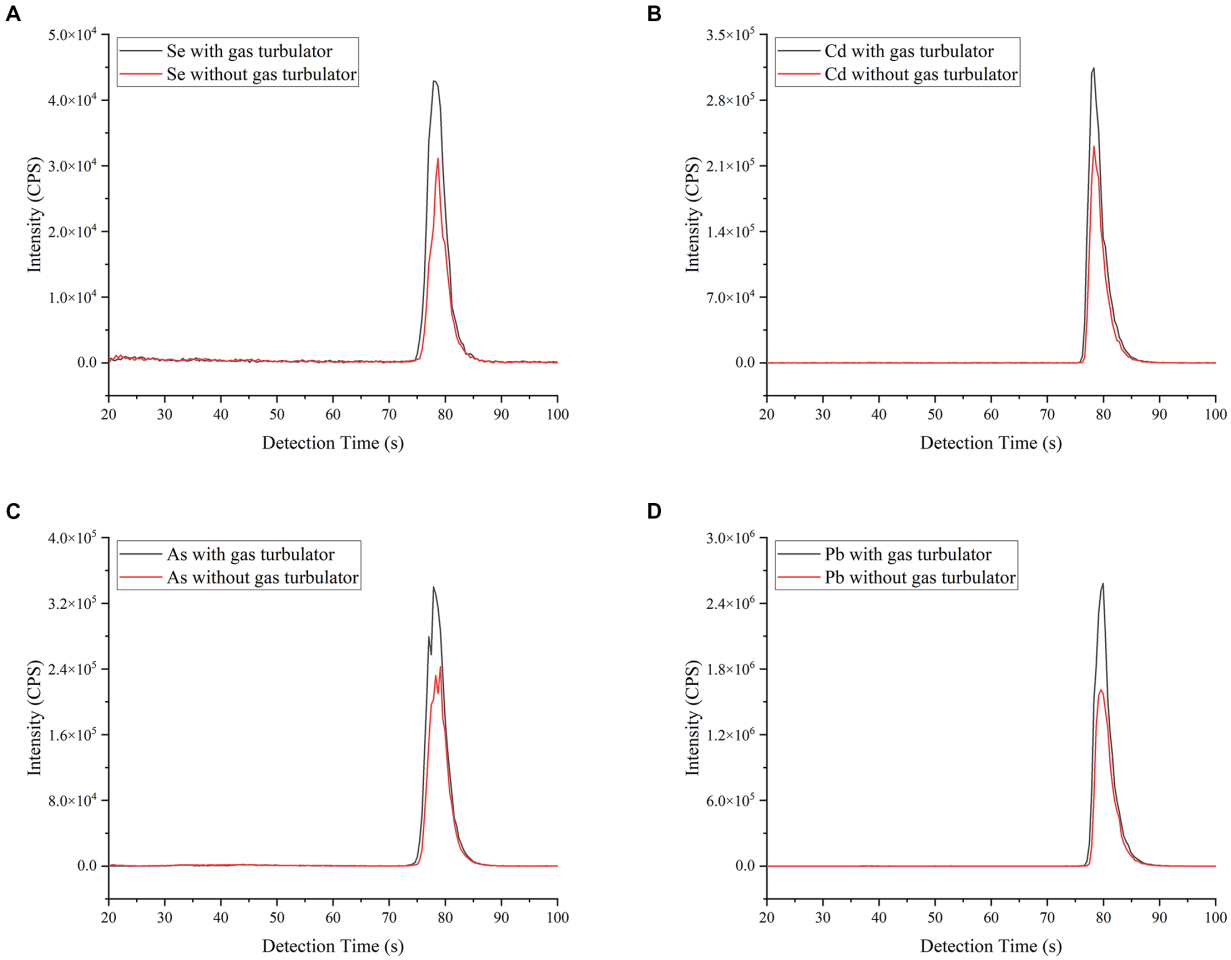
Effect of the gas turbulator line design on ICP-MS signals of Se, Cd, As and Pb. The other operating parameters are consistent with Supplementary Table S1. A soybean CRM (GBW10013) sample (As = 35 ± 12 ng g^−1^; Se = 22 ng g^−1^; Cd = 11 ng g^−1^ and Pb = 70 ± 20 ng g^−1^) was employed for slurry sampling. **(A–D)** refer to signal peak, with or without gas turbulator, of Se, Cd, As and Pb, respectively.

In addition, the effect of gas turbulator design on the signal intensity of ETV-ICP-MS was investigated using a soybean CRM (GBW10013) sample, and the results are shown in Figure 2. With 600 mL min^**−1**^ Ar gas turbulator, the ICP-MS signal intensities of Se, Cd, As and Pb all show an increase for whatever peak area or height vs. without gas turbulator, of which the increase rate ranged from 28 to 63% based on peak area. This result might attributed to the effective improvement of analyte transportation and the alleviation of memory effect caused by gas turbulator. As a result, the following studies were carried out using the gas turbulator line.

#### 3.1.2. Signal delay device (SDD)

**Figure 3:**
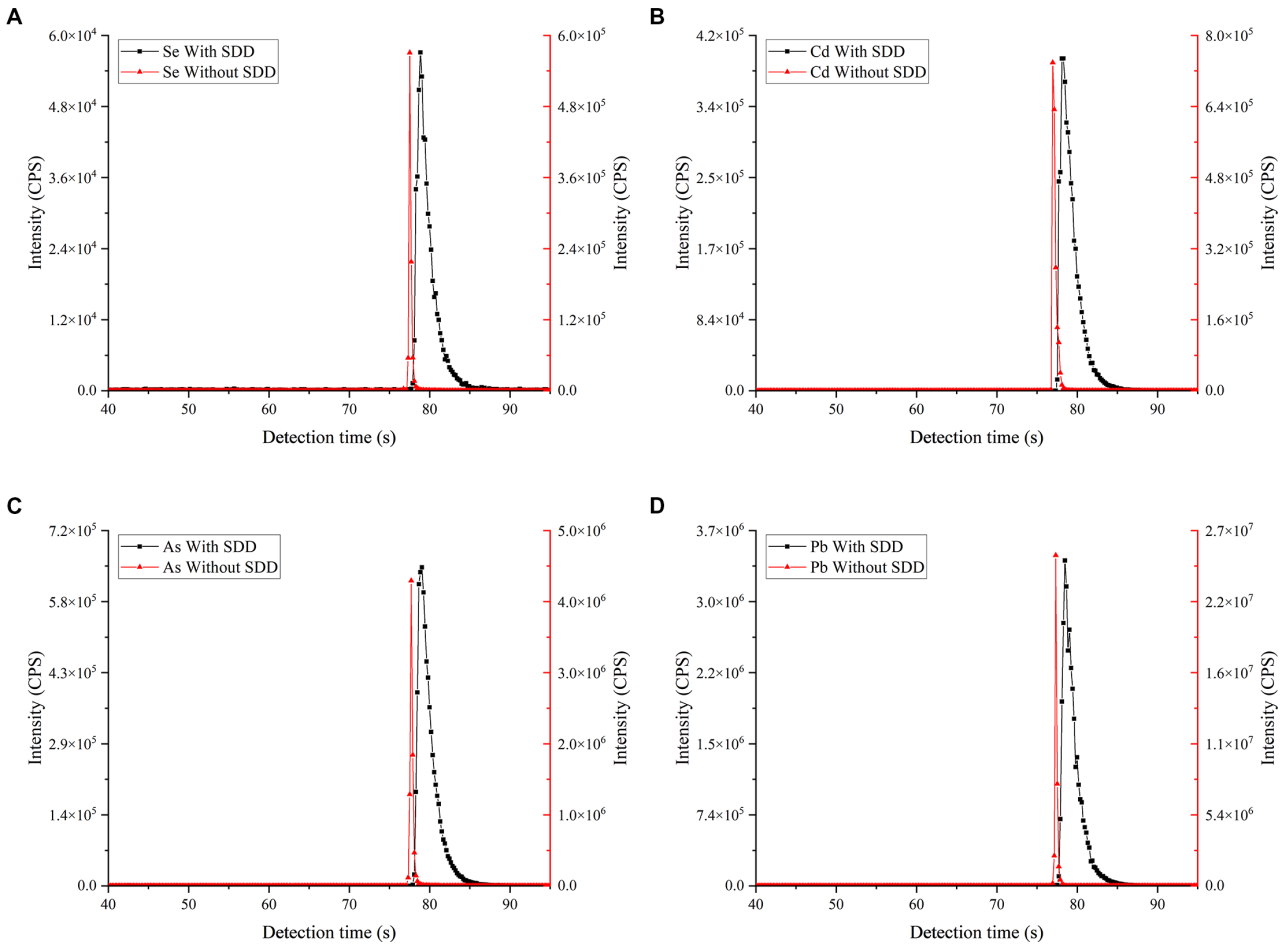
The comparison of Se, Cd, As and Pb signal peaks with or without SDD. The operational parameters were consistent with Supplementary Table S1. A soybean CRM (GBW10013) sample (As = 35 ± 12 ng g^−1^; Se = 22 ng g^−1^; Cd = 11 ng g^−1^ and Pb = 70 ± 20 ng g^−1^) was employed for slurry sampling **(A–D)** refer to signal peak, with or without SDD, of Se, Cd, As and Pb, respectively.

Compared with the continuous liquid sampling mode via peristaltic pump, ETV renders a transient analyte vaporization (lasting for only several seconds) for signal acquisition by ICP-MS. Under the optimized instrumental conditions of ICP-MS (SUPEC 7000), approximately 10 or more signal points can be acquired during the total run time of one vaporization cycle. Obviously, these signal points enable forming an individual peak, however, such a small amount of acquisition points would lose partial signal information, thereby resulting in an adverse impact for analytical precision (31, 32). To solve this problem, a signal delay device (SDD) made of PFA was employed as a cyclonic nebulizer to connect the graphite furnace with ICP-MS. When the vaporized analyte was transported into the SDD, a cyclone formed due to the inner circling structure to remain matrix micro-particles herein; as well, the inner capacity (~40 mL) plays a dilution effect and results in signal delay from 2 seconds to 5 seconds as shown in Figure 3. As a result, signal point number for the total run time was increased from 8 to 35, which is beneficial to obtain a more complete signal peak and a more stable analysis with regard of the effect of matrix particle interference. To verify this conclusion, a slurry rice sample was employed for repeated measurement (*n* = 6) by ETV-ICP-MS with or without SDD; the relative standard deviations (RSD) imply a significant improvement from 14.8–16.2% to 1.7–2.5%, revealing a better analytical precision for using SDD. Of course, the absolute analytical sensitivities (peak width at half height) of ETV-ICP-MS become worse due to the SDD’s dilution effect as shown in Figure 3; while, the peak area at this moment can be obviously increased. In fact, this current LODs of ETV-ICP-MS are competent to the determination of Se, Cd, As and Pb in real food samples. As a compromise, this SDD design was chosen for this ETV-ICP-MS instrumentation.

#### 3.1.3. Signal acquisition of ICP-MS

For the signal acquisition of ICP-MS instrumentation, the run time of one point is commonly composed of setting time and dwell time; and the former one depends on instrumental electronic components, which is always constant for an ICP-MS setup, eg. the setting time of ICP-MS used in this work was ~15 ms. So, only the dwell time was optimized here; as well, the dwell time should be minimized as possible. According to the adjustable parameter of this ICP-MS setup, 10 ms to 30 ms of the dwell times were set and their effects were investigated in Supplementary Figure S3. Herein, changing dwell time indicates no obvious impact on the signal intensity (peak height). However, the RSDs (*n* = 6) at 10 ms, 20 ms, and 30 ms were 1.2–8.1%, 4.5–8.8%, and 8.5–18.2% for the dwell time, respectively. Finally, the dwell time was set as 10 ms for the following experiments.

### 3.2. Sample dehydration and ashing

**Figure 4:**
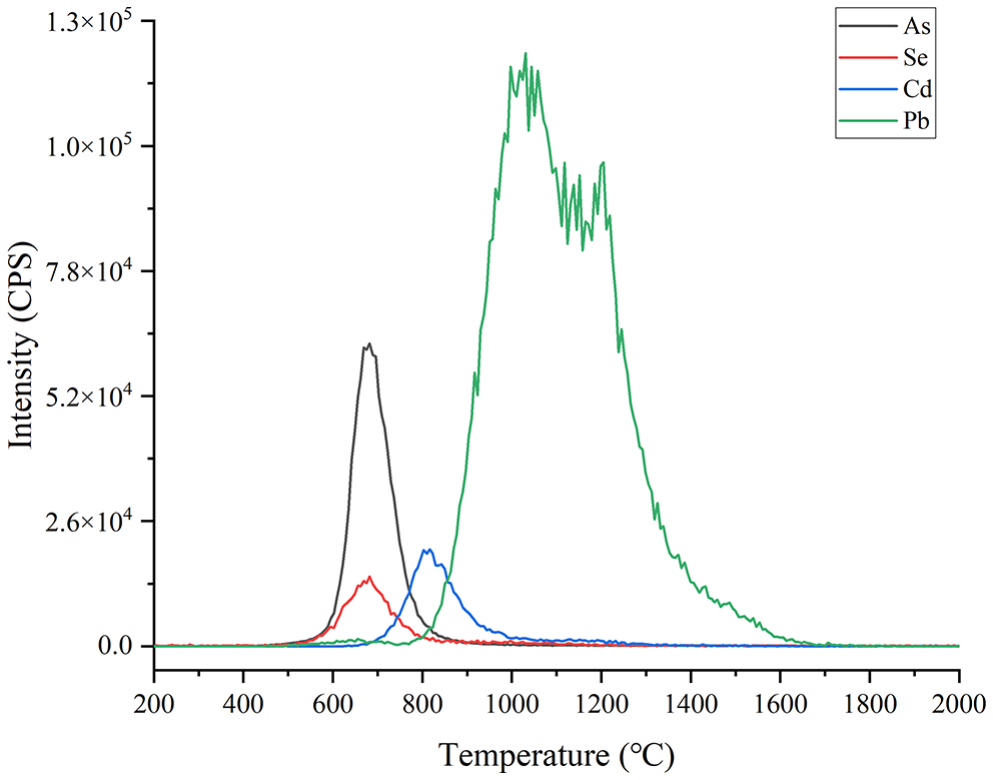
Se, Cd, As and Pb signal peaks with the increase of ETV temperature. The operating parameters of ETV monitoring are shown in Supplementary Table S5. A soybean CRM sample (GBW10013, As = 35 ± 12 ng·g^−1^; Se = 22 ng g^−1^; Cd = 11 ng  g^−1^ and Pb = 70 ± 20 ng g^−1^) was employed for slurry sampling. The temperature was controlled to rise from room temperature to 2000°C in 55 s.

The composition of food matrix is complicated including water, carbonhydrate, protein, lipid and so on, which are always a puzzle for the direct slurry sampling elemental analysis. In theory, these potential interferents mentioned above should be removed as possible prior to vaporization, as well as avoiding the loss of the targeted analytes of Se, Cd, As and Pb. As usual, the dehydration and ashing via heating must be indispensable to remove moisture and organic substances to separate with targeted elements. To investigate the heating conditions, a soybean CRM (GBW10013) sample was measured under different temperatures of ETV. As shown in Figure 4, from 500°C, As and Se start to be vaporized and finish before 900°C; Cd is completely vaporized from 700 to 1,000°C; and Pb ranges from 800 to 1700°C, which are almost consistent with the previous studies (33, 34). To avoid the loss of As and Se, the highest temperature of dehydration and ashing must be controlled below 500°C. Herein, 450°C was proved effective for ashing food samples including grain, oilseed, tea, and celery, spinach, carrot. At the same time, to avoid the boiling-over loss of slurry sample caused by overheating, the dehydration and ashing should be performed with a mild heating process. As a compromise, the dehydration process was completed from room temperature to 200°C within 20 s and held for 15 s, and then continued to heat to 450°C within 20 s and then held for 20 s for sample ashing. In addition, the carrier and gas turbulatores were set as 100 mL min^**−1**^ and 300 mL min^**−1**^, respectively, to blow the moisture, gaseous interferents and microparticles out of the ETV and prevent them from entering the SDD and ICP-MS.

### 3.3. Vaporization for different elements

After ashing, the targeted elements remained in sample residue and got ready for the following vaporization under high temperature. From Figure 4, 1800°C heating fulfilled the complete vaporization of Se, Cd, As and Pb. Thus, to ensure the complete vaporization of all targeted elements in various food matrices, the highest vaporization temperature was set as 2,300°C and the heating process was finished within 1 s under the optimized power supply condition. The following results also proved 2,300°C was competent for the complete vaporization of Se, Cd, As and Pb from various plant food samples.

### 3.4. Working gas

Also, the gas line system plays a key role in the transportation of analyte aerosol to the following SDD and ICP-MS, including carrier gas and gas turbulator herein. To investigate the effect of carrier gas and gas turbulator, a rice CRM sample was measured under different gas flow rates, and the results are shown in Supplementary Figure S4. The signal intensities of Se, Cd, As and Pb reached the highest values under 500 mL min^**−1**^ carrier gas and 600 mL min^**−1**^ gas turbulator with favorable RSDs in the range of 1.1–2.6%. As well, the total flow rate of carrier and gas turbulatores was 1,100 mL min^**−1**^ and is able to replace the nebulizing gas for ICP-MS pneumatic nebulization. However, excessive gas flow could dilute the analyte aerosol to thereby reduce the signal intensities. Therefore, the argon carrier and gas turbulatores were chosen as 500 mL min^**−1**^ and 600 mL min^**−1**^, respectively.

### 3.5. Interference study

To further investigate the possible interference of inorganic elements, 10 μL of solutions containing Na, Mg, K, Fe, Ca, Co, Mo, etc. were separately mixed with 10 μL of a mixture of Se, Cd, As and Pb (20 ng mL^**−1**^) for measurement. Since these elements that may cause interference due to their high content in the matrix of food samples. As shown in Table 1, the average recoveries of Se, Cd, As and Pb ranged from 91.5 to 112.5%, indicating free of significant interference at normal elemental levels in food matrices.

**Table 1:** The interference of potential substances (*n* = 3).

Substances	Added^a^	Se		Cd		As		Pb	
	(mg L^−1^)	Recovery^b^ (%)	CV%	Recovery^b^ (%)	CV%	Recovery^b^ (%)	CV%	Recovery^b^ (%)	CV%
P	1%^c^	97.2	2.8	103.0	3.0	98.1	1.9	107.9	7.9
S	1%^c^	99.6	0.4	106.1	6.1	100.3	0.3	109.5	9.5
Na	100	109.5	9.5	111.3	11.3	98.4	1.6	91.5	8.5
Mg	100	104.3	4.3	108.4	8.4	107.5	7.5	100.4	0.4
K	100	98.0	2.0	112.5	12.5	97.1	2.9	95.8	4.2
Zn	100	100.8	0.8	100.9	0.9	101.2	1.2	102.9	2.9
Cu	10	111.3	11.3	108.1	8.1	109.1	9.1	104.6	4.6
Mn	10	102.7	2.7	106.1	6.1	103.2	3.2	109.5	9.5
Fe	10	109.4	9.4	103.8	3.8	107.9	7.9	93.5	6.5
Ca	10	107.3	7.3	103.8	3.8	107.6	7.6	96.2	3.8
Co	1	107.5	7.5	107.2	7.2	107.7	7.7	93.8	6.2
Mo	1	108.2	8.2	99.3	0.7	103.2	3.2	93.6	6.4

a A series of 10 μL of Se, Cd, As and Pb standard solutions containing a substance at the above added levels were measured using the proposed method.

b Recovery (%) is: measured concentration/added concentration × 100%.

c *p* and *S* were added at 1% (v:v).

However, as shown in Supplementary Figure S5, the slope of the calibration curve using standard additions is significantly higher than the slope of the standard solution calibration. So, when introducing real food samples such as rice, tea and spinach, there is still some matrix interference, which are possibly caused by the “carry on” effect of microparticles accompanied with targeted elements (35, 36). These matrix interferents are detrimental to the analytical accuracy of the proposed ETV-ICP-MS method. To solve this problem, the calibration strategy of standard addition method was used to calibrate Se, Cd, As, and Pb in food samples (37). Under the above conditions, the linearity *R^2^* of each element was at least 0.999, proving that standard addition method is competent here considering the results of linearity.

### 3.6. Analytical performances and real food sample analysis

Under optimized conditions, the limits of detection (LOD) were calculated for 11 assays of Se, Cd, As and Pb measurements in 10 μL Triton X-100 (0.5%, v:v) using an equation 3 σ/m (σ is the standard deviation and m is the slope of the Se, Cd, As and Pb calibration curves); and the limits of quantification (LOQ) using an equation 10 σ/m (38). The method LODs were 0.5 ng g^−1^, 0.3 ng g^−1^, 0.3 ng g^−1^ and 0.6 ng g^−1^ for Se, Cd, As and Pb, respectively; the corresponding LOQs were 1.7 ng g^−1^, 1.0 ng g^−1^, 1.0 ng g^−1^ and 1.9 ng g^−1^, respectively; and the linearity *R^2^* > 0.999 was reached in the range of 1 to 4,000 ng g^**−**1^ using the standard addition method.

To verify the accuracy of the method, real food samples including CRM like rice, tea and soybeans (n = 6) were measured using this proposed method and microwave digestion ICP-MS method or directly compare the certified values. As shown in the Table 2, the results were in agreement with the certified value of CRMs and that of microwave digestion ICP-MS; the recoveries (the recovery was calculated as “the value measured by this method/the value measured by the standard method or the certified value “× 100%) of multi-elements in food samples were 86–118%, and the RSDs (*n* = 6) of repeated measurements were in the range of 1.2–8.9%. These results demonstrate a favorable analytical precision and accuracy of Se, Cd, As and Pb in typical food sample matrices mentioned above; as well, the analysis can be completed within 3 min only using a simple sample preparation, providing a fast, green and robust instrumental method. To compare the analytical performances, the related ETV-ICP-MS methods for food analysis previously reported (17, 32, 34, 38, 40–43)are summarized in Supplementary Table S6. The LODs of Se, Cd, As and Pb in this work was lower than or comparable to that of other ETV-ICP-MS methods previously reported; as well, the analytical precision is better. Furthermore, this proposed method is applicable to more food samples including carbonhydrate, protein, lipid, fibre-rich matrices. So, the novel disgn of SDD with gas turbulator is more beneficial for ETV-ICP-MS application.

**Table 2:** Elemental presence and recovery in food samples by the proposed method (*n* = 6).

Samples^a^	Se				Cd				As				Pb			
	Standard method (ng g^−1^)	This method (ng g^−1^)	Recovery (%)	CV% (%)	Standard method (ng g^−1^)	This method (ng g^−1^)	Recovery (%)	CV% (%)	Standard method (ng g^−1^)	This method (ng g^−1^)	Recovery (%)	CV% (%)	Standard method (ng g^−1^)	This method (ng g^−1^)	Recovery (%)	CV% (%)
Spinach	90 ± 8	100 ± 2	111	11	382 ± 5	357 ± 10	93	7	143 ± 5	141 ± 5	99	1	276 ± 12	312 ± 14	113	13
Celery	176 ± 7	163 ± 6	93	7	125 ± 3	142 ± 8	114	14	358 ± 27	310 ± 22	87	13	610 ± 22	594 ± 25	97	3
Carrot	43 ± 3	38 ± 3	88	12	31 ± 1	34 ± 2	110	10	90 ± 15	87 ± 3	97	3	336 ± 2	288 ± 22	86	14
Soybean powder (GBW10013^b^)	(22)	24 ± 1	109	9	(11)	10 ± 1	91	9	35 ± 12	34 ± 3	97	3	70 ± 20	74 ± 4	106	6
Tea powder (GBW10016a^b^)	90 ± 40	88 ± 7	98	2	46 ± 5	49 ± 3	107	7	100 ± 20	93 ± 4	93	7	1,090 ± 130	1,142 ± 51	105	5
Tea powder (GBW10052a^b^)	90 ± 30	106 ± 2	118	18	200 ± 20	194 ± 3	97	3	160 ± 20	146 ± 11	91	9	1,600 ± 200	1791 ± 50	112	12
Rice powder (GBW10010a^b^)	36 ± 8	36 ± 2	100	0	53 ± 4	57 ± 1	108	8	80 ± 10	88 ± 4	110	10	100 ± 20	112 ± 2	112	12
Rice powder (GBW10045a^b^)	60 ± 10	55 ± 1	92	8	320 ± 40	305 ± 12	95	5	120 ± 20	135 ± 2	113	13	80 ± 20	93 ± 3	116	16

a For each sample, 10 μL of the prepared slurry sample was measured.

b CRMs show certified uncertainties in Se, Cd, As and Pb concentrations that can be compared with the results of this method.

To compare this proposed method with the standard method, their analysis conditions and cost were listed as shown in Supplementary Table S7. This proposed method is free of strong acid, and hazardous chemicals and only consumes 8 min for one aliquot including sample preparation vs. > 8 h using the standard method of wet digestion coupled with ICP-MS. At the same time, the “green” performance of the method was evaluated using the new metric software “AGREE” (39). Twelve parameters such as sample processing, sample size, and energy consumption were evaluated and a score was obtained. From Supplementary Table S7, the score of the standard method is 0.47 and the score of this method is 0.68. Therefore, this method is relatively greener compared with the standard method. By the way, some problems in this work should be solved in the future, eg. using the solution standard calibration to replace the standard addition method, and detecting more elements in more food sample matrices.

## 4. Conclusion

In this work, a novel slurry sampling analyzer was developed for the first time for the direct and simultaneous determination of trace Se, Cd, As and Pb in plant food samples including grain, oilseed, celery, spinach, carrot and tea. The signal delay device coupled with a gas turbulator line system were first designed to improve the sensitivity and precision of the analysis for ETV-ICP-MS, respectively. As a result, the analytical precision (RSDs) was improved from 14.8–16.2% (without SDD) to 1.2–8.9% (with SDD) in real food samples. Further, the method LODs for Se, Cd, As and Pb were 0.5 ng g^−1^, 0.3 ng g^−1^, 0.3 ng g^−1^ and 0.6 ng g^−1^ for 10 μL slurry samples, respectively. The LOQs for Se, Cd, As and Pb were 1.7 ng g^−1^, 1.0 ng g^−1^, 1.0 ng g^−1^ and 1.9 ng g^−1^, respectively. For the new DSS-ETV-ICP-MS instrumentation, the whole analysis time was less than 8 min without sample digestion process. This proposed direct slurry sampling ICP-MS method is thus suitable for rapid and sensitive determination of Se, Cd, As and Pb in food samples with advantages such as simplicity, green and safety, as well as with a promising application potential in detecting more elements to protect food safety and human health.

## Data availability statement

The original contributions presented in the study are included in the article/Supplementary material, further inquiries can be directed to the corresponding authors.

## Author contributions

GL, JY, XY, XL, and XM designed the research and provided the data. GL, QL, and JY performed the statistical analyses. QL, XL, and XM wrote the paper. CQ, JY, and XM re-viewed and edited the manuscript. GL, XL, CQ, JY, and XM had primary responsibility for the final content. All authors contributed to the article and approved the submitted version.

## Funding

This work was funded by the Scientific and Technological Project of Tibet Autonomous Region (XZ202001ZY0048N), National Natural Science Fund of China (No. 32072307), Key Research and Development Project of Hainan Province (No. ZDYF2021XDNY185) and Modern Agricultural Industry Technology System (No. CARS-05). As well, sincerest thanks to the support of the Agricultural Science and Technology Innovation Program presided by XM.

## Conflict of interest

JY and XY were employed by Hangzhou Puyu Technology Co. Ltd.

The remaining authors declare that the research was conducted in the absence of any commercial or financial relationships that could be construed as a potential conflict of interest.

## Publisher’s note

All claims expressed in this article are solely those of the authors and do not necessarily represent those of their affiliated organizations, or those of the publisher, the editors and the reviewers. Any product that may be evaluated in this article, or claim that may be made by its manufacturer, is not guaranteed or endorsed by the publisher.

## Supplementary material

The Supplementary material for this article can be found online at: https://www.frontiersin.org/articles/10.3389/fnut.2023.1201801/full#supplementary-material
