# Supplementary material for: Rapid and sensitive determination of Se and heavy metals in foods using electrothermal vaporization inductively coupled plasma mass spectrometry with a novel transportation system
Source: Front Nutr. 2023 Jun 7;10:1201801. doi: 10.3389/fnut.2023.1201801 (PMC10282127; doi:10.3389/fnut.2023.1201801)
Supplement: Supplementary file 1 [file Data_Sheet_1.docx]

Supplementary Material

Rapid and sensitive determination of Se and heavy metals in foods using electrothermal vaporization inductively coupled plasma mass spectrometry with a novel transportation system

Guanyu Lan^1,2#^, Xue Li^2#^, Jijun Yao^3#^, Xiaofeng Yu^3^, Qinghai Liu^1^, Cheng Qiu^1*^, and Xuefei Mao^2*^

**#Lan, Li, and Yao contribute equally to this work.**

*** Correspondence:** Cheng Qiu: [chengqiu_2006@163.com](mailto:chengqiu_2006@163.com); Xuefei Mao: mxf08@163.com & [maoxuefei@caas.cn](mailto:maoxuefei@caas.cn)

**Table S-1 Instrumental program of ETV system**

| **Procedure** | **ETV temperature**  **(**°C**)** | **Heating time**  **(s)** | **Holding time**  **(s)** | **Carrier gas**  **(mL min^-1^)** | **Gas turbulator**  **(mL min^-1^)** | **Signal acquisition** |
| --- | --- | --- | --- | --- | --- | --- |
| Dehydration | 200 | 20 | 15 | 100 | 300 |  |
| Ashing | 450 | 20 | 20 | 100 | 300 |  |
| Vaporization | 2300 | 1 | 5 | 500 | 600 | √ |
| Detection |  |  |  |  |  |  |
| Cleaning | 2500 | 1 | 7 | 500 | 800 |  |

**Table S-2 Instrumental program of ICP-MS**

| **Instrument parameters** | **Setting value** |
| --- | --- |
| RF Power/ (W) | 1400 |
| Auxiliary flow/ (L min^-1^) | 1 |
| Cooling gas flow/ (L min^-1^) | 14 |
| Sampling depth/ (mm) | 2 |
| Sampling cone/interception cone | Ni/Cu |
| Scanning times | 1 |
| Scan mode | peak hopping scan |
| Dwell time/ (ms) | 10 |
| Isotopes | ^75^As, ^82^Se, ^111^Cd, ^208^Pb |

**Table S-3 Microwave digestion operating conditions.**

| **Heating time(min)** | **Temperature (℃)** | **Holding time (min)** |
| --- | --- | --- |
| 5 | 150 | 3 |
| 5 | 200 | 3 |
| 8 | 240 | 30 |

**Table S-4 ICP-MS conditions for the detection of digested samples.**

| **Instrument parameters** | **Setting value** |
| --- | --- |
| RF power/ (W) | 1550 |
| Nebulization gas flow/ (L min^-1^) | 1.150 |
| Auxiliary gas flow/ (L min^-1^) | 1.00 |
| Cooling gas flow/ (L min^-1^) | 14.00 |
| Sampling depth/ (mm) | 2 |
| Sampling cone/interception cone | Ni/Cu |
| Scanning times | 10 |
| Dwell time/ (ms) | 30 |
| Scan mode | peak hopping scan |
| Collision gas | He |
| Collision gas flow/ (mL min^-1^) | 1.38 |
| Isotope | ^75^As, ^82^Se, ^111^Cd, ^208^Pb |
| Internal standard | ^103^Rh、^115^In、^185^Re、^209^Bi |

**Table S-5** **Heating program of ETV for As, Se, Cd and Pb vaporization temperature study**

| **ETV temperature (℃)** | **Heating time (s)** | **Holding time (s)** | **Carrier gas flow (mL min^-1^)** | **Gas turbulator flow (mL min^-1^)** | **Reading** |
| --- | --- | --- | --- | --- | --- |
| 2000 | 55 | 0 | 500 | 600 | √ |

**Table S-6 Comparison of analytical performances for Se, Cd, As and Pb by different ETV-ICP-MS methods in literature.**

| **Methods** | **Analyte** | **LODs**  **(ng g^-1^)** | **RSD**  **(%)** | **Time**  **(min)** | **Sample matrix** | **Sampling method** | **Ref.** |
| --- | --- | --- | --- | --- | --- | --- | --- |
| ETV-DRC-ICP-MS | Cr, Cd, Hg, Pb | 0.1-0.6 | ＜19% | ~3 | Honey | Slurry sampling | (34) |
| USS-ETV-DRC-ICP-MS | Cr, Cu, Cd, Hg, Pb | 0.4-1.7 | - | ~2.5 | Rice flour | Slurry sampling | (17) |
| USS-ETV-ICP-MS | Cd, Hg, Pb | 3-9 | ＜14% | ~3 | Fish | Slurry sampling | (32) |
| USS-ETV-ICP-MS | Ge, As, Se, Hg | 7-34 | - | ~3.5 | Fish | Slurry sampling | (40) |
| TBF-ETV-ICP-MS | Pb | 10.2 | 6.5% | ~3 | Serum | Solid sampling | (41) |
| USS-ETV-ICP-MS | Zn, As, Cd, Sb, Hg, Pb | 0.1-3.6 | ＜10% | ~2 | Serum | Slurry sampling | (42) |
| ETV-TCT-ICP-MS | Zn, Cd | 0.01-1 | 4.7-7.9% | ~2.5 | Grain | Solid sampling | (38) |
| HF-LPME-ETV-ICP-MS | Cu, Zn, Pd, Cd, Hg, Pb, Bi, | 1.6-28.7 | 6.1-10.8% | ~1 | Peach leaves, sea water | Slurry sampling | (43) |
| ETV-ICP-MS | As, Se, Cd, Pb | 0.3-0.6 | 1.2-8.9% | ~3 | Grain, oilseed, vegetables, tea | Slurry sampling | This work |

**Table S-7 Comparison of analytical performances this method with the standard method(single analysis).**

|  | This method | Microwave digestion ICP-MS method |
| --- | --- | --- |
| LODs (ng g^-1^) | 0.3~0.6 | 0.002~0.089 |
| LOQs (ng g^-1^) | 1.0~1.9 | 0.006~0.296 |
| RSDs (%) | 1.2~8.9 | 1.2~12.3 |
| Required reagents | 1 mL Triton X-100(0.5%, *v:v*) | 1 mL DI water; 2mL HNO_3_; 1mL H_2_O_2_; 15mL HNO_3_(2%, *v:v*) |
| Total sample analysis time (including sample analysis time and sample preparation time, min) | 8 | 65 |
| Sample size (g) | 0.05 | 1 |


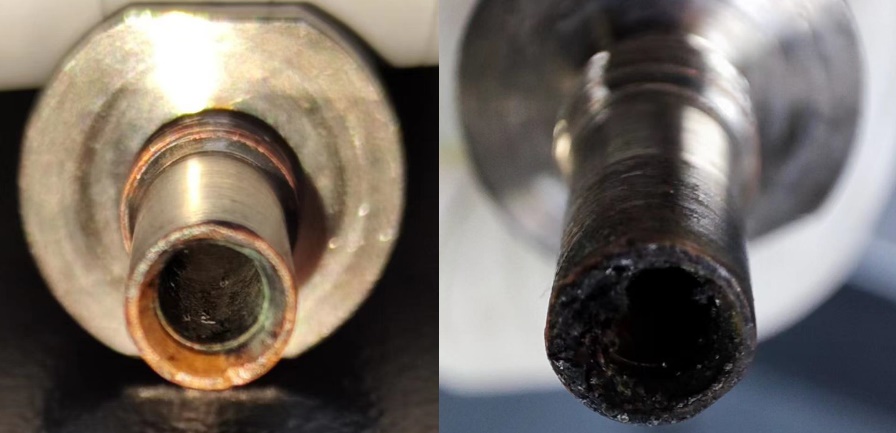


**Figure S-1 Effect of gas turbulator on residual deposition on the pagoda joint.** The picture on the left shows the pagoda joint with gas turbulator after sampling 50 replicates, and the inner wall is smooth and there is no obvious residual deposition. The picture on the right shows the pagoda joint without gas turbulator after sampling 50 replicates, and the residual deposition was obviously found.


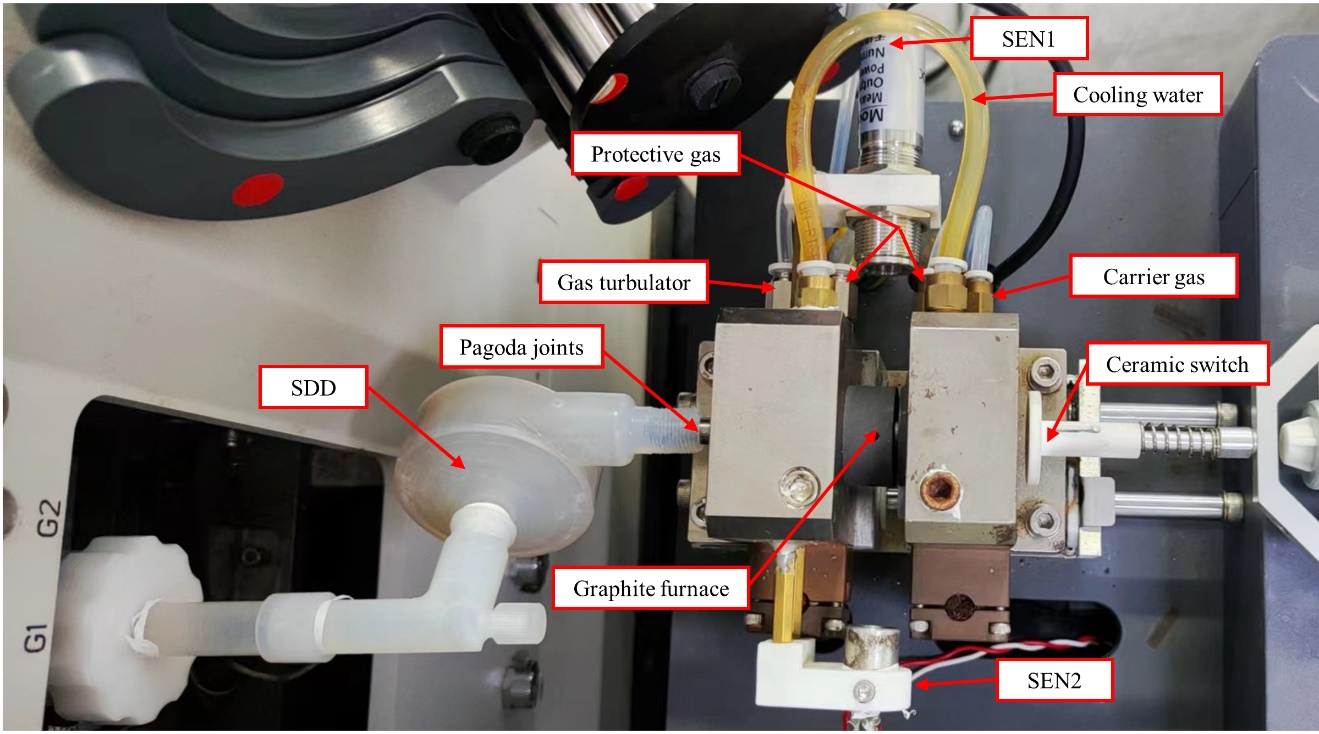


**Figure S-2** **Picture of this proposed ETV-ICP-MS instrumentation.** SEN1 and SEN2 refer to the photoelectric sensor and infrared sensor, respectively. SSD refers to the signal delay device.

**
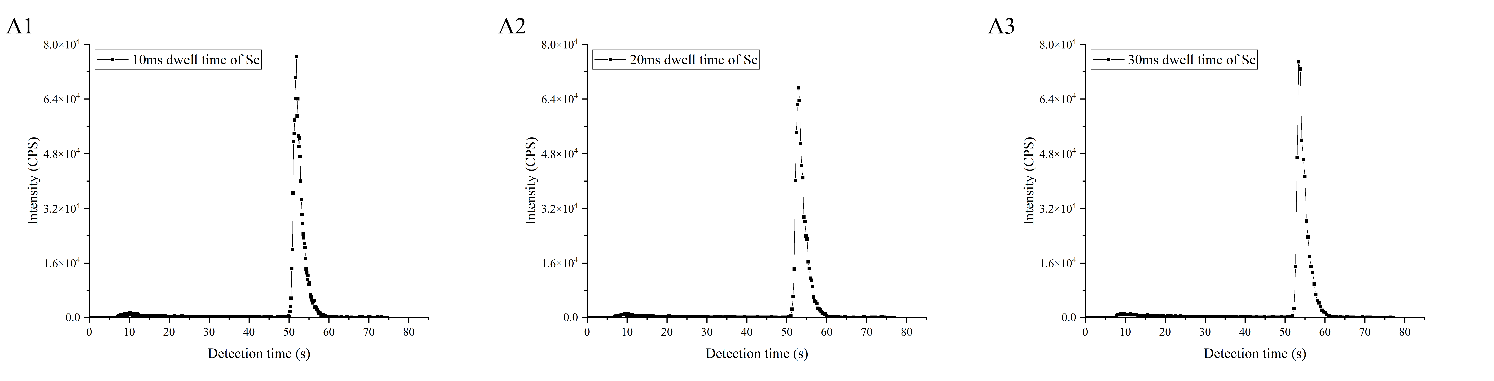
**

**
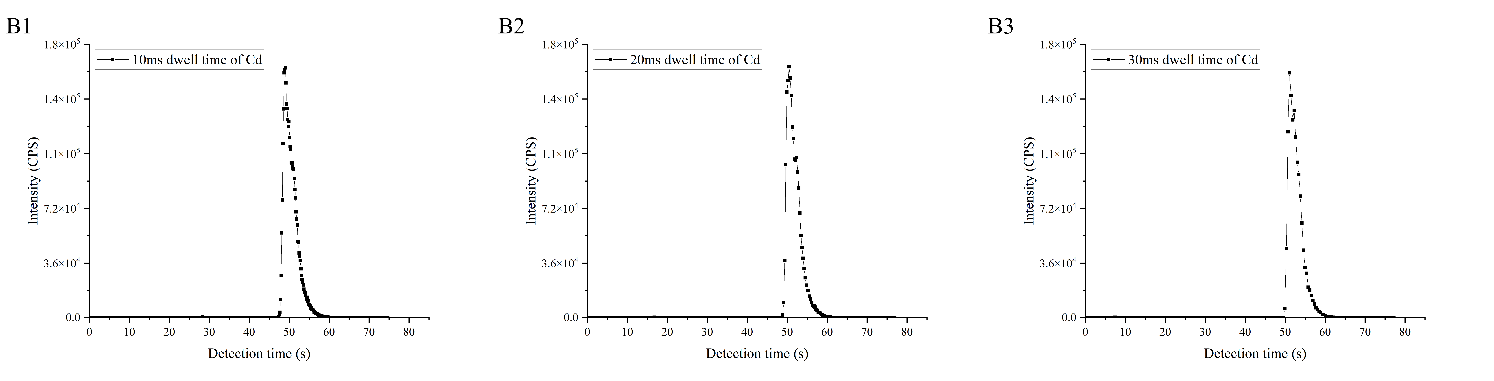
**

**
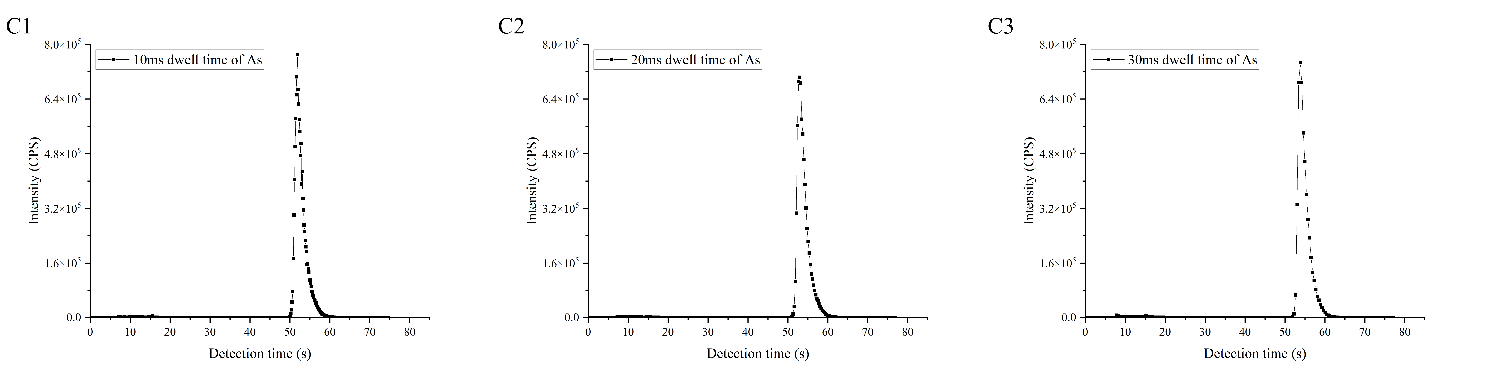
**

**
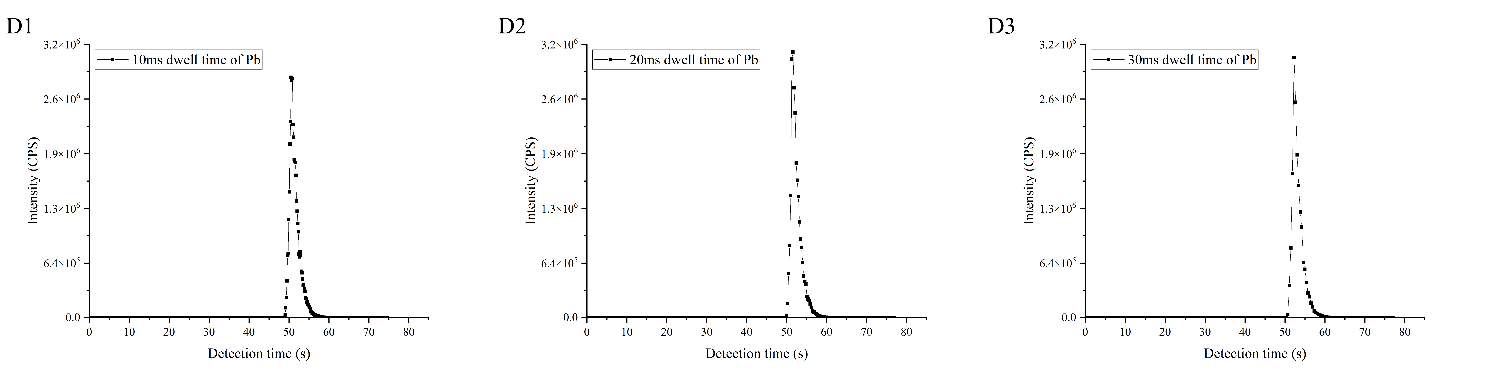
**

**Figure S-3 The comparison of As, Se, Cd and Pb signal peaks using different dwell times.** The operational parameters were consistent with Table 1. A rice sample (GBW10010a, As = 80 ± 10 ng g^-1^; Se = 36 ± 8 ng g^-1^; Cd = 53 ± 4 ng g^-1^ and Pb = 100 ± 20 ng g^-1^) was employed for slurry sampling. A1, A2 and A3 refer to the peak shapes of Se at 10, 20, 30 dwell time. B1, B2 and B3 refer to the peak shapes of Cd at 10, 20, 30 dwell time. C1, C2 and C3 refer to the peak shapes of As at 10, 20, 30 dwell time. D1, D2 and D3 refer to the peak shapes of Pb at 10, 20, 30 dwell time.

**
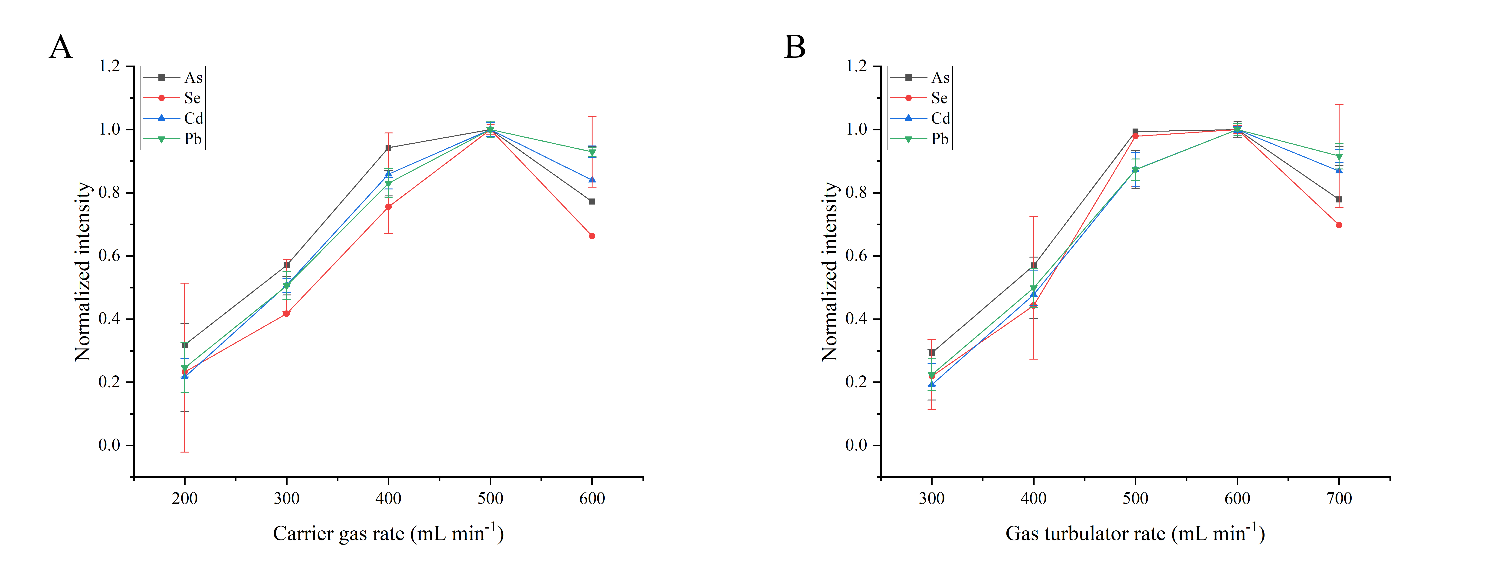
**

**Figure S-4. The effect of carrier and gas turbulatores on ICP-MS signal intensities of Se, Cd, As and Pb.** The other operational parameters are consistent with **Table 1**. The number of repetitions of the error bars was 3 times. A rice CRM sample (GBW 10045a, As = 120 ± 20 ng g^-1^; Se = 60 ± 1 ng g^-1^; Cd = 320 ± 40 ng g^-1^ and Pb = 80 ± 20 ng g^-1^) was employed for slurry sampling. A refers to the carrier gas rate, B refers to the gas turbulator rate.

**
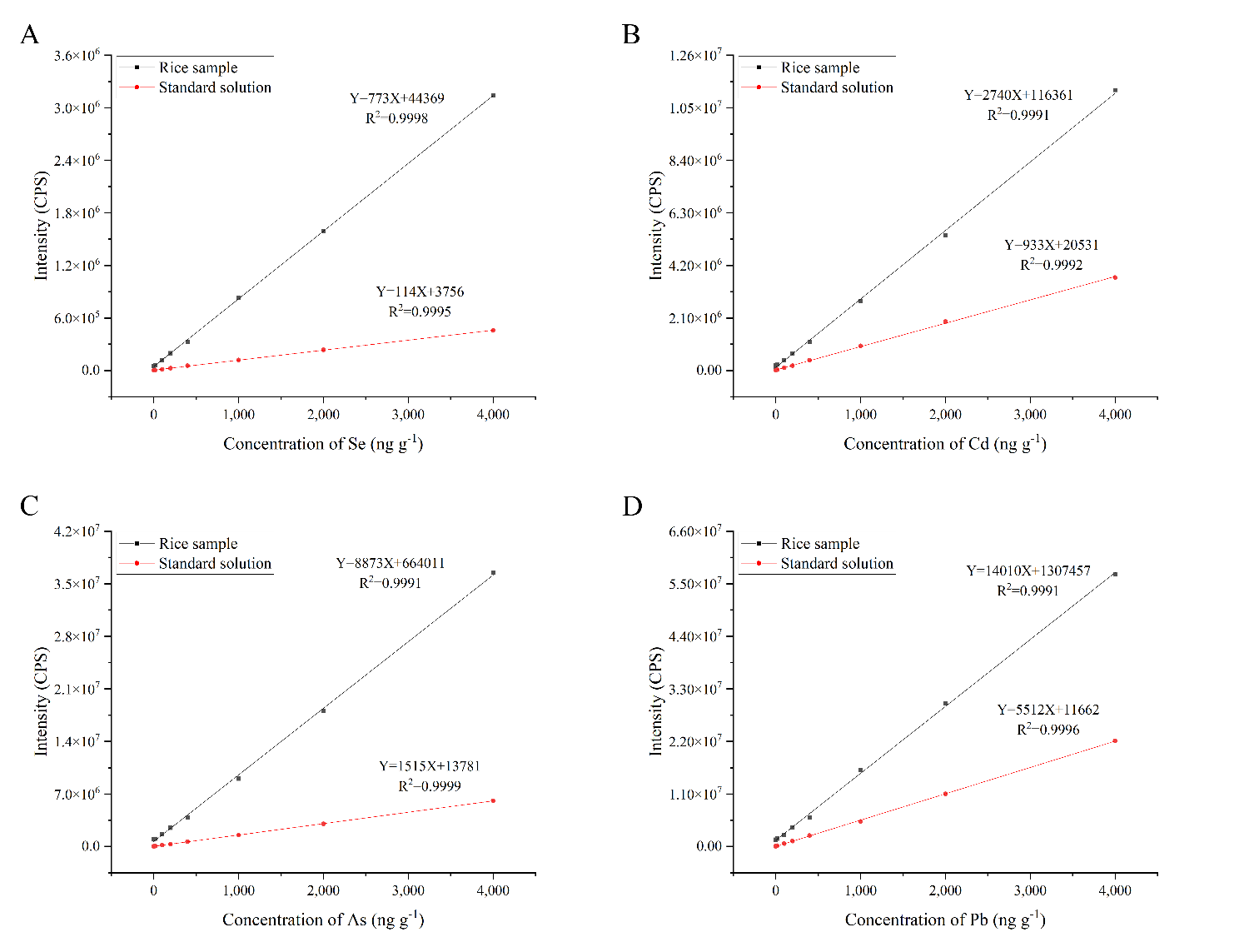
Figure S-5 The calibration curves of Se, Cd, As and Pb with standard addition *vs.* standard solution.** The operational parameters were consistent with **Table 1**. For the standard addition method, a rice sample (GBW10010a, As = 80 ± 10 ng g^-1^; Se = 36 ± 8 ng g^-1^; Cd = 53 ± 4 ng g^-1^ and Pb = 100 ± 20 ng g^-1^) was employed for slurry sampling and spiked by 1, 20, 100, 200, 400, 1000, 2000 and 4000 ng g^-1^ of Se, Cd, As and Pb. For the standard solution method, 1, 20, 100, 200, 400, 1000, 2000 and 4000 ng g^-1^ Se, Cd, As and Pb were introduced into the ETV for measurement using 10 μL standard solution. A, B, C, D refer to the Se, Cd, As and Pb, respectively.


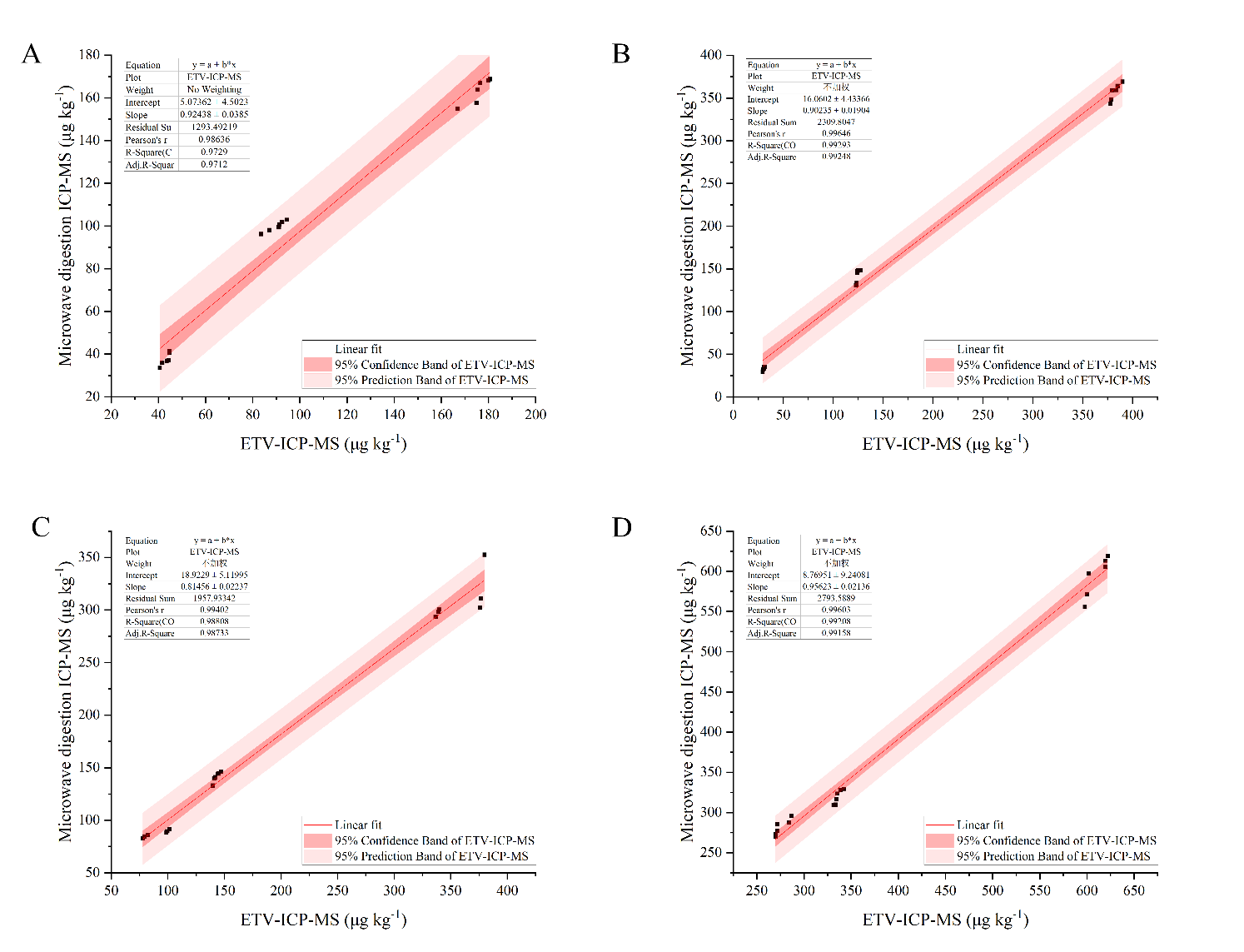


**Figure S-6 Comparison of Se, Cd, As and Pb concentrations measured by ETV-ICP-MS and microwave digestion ICP-MS.** A, B, C, D refer to the Se, Cd, As and Pb, respectively.


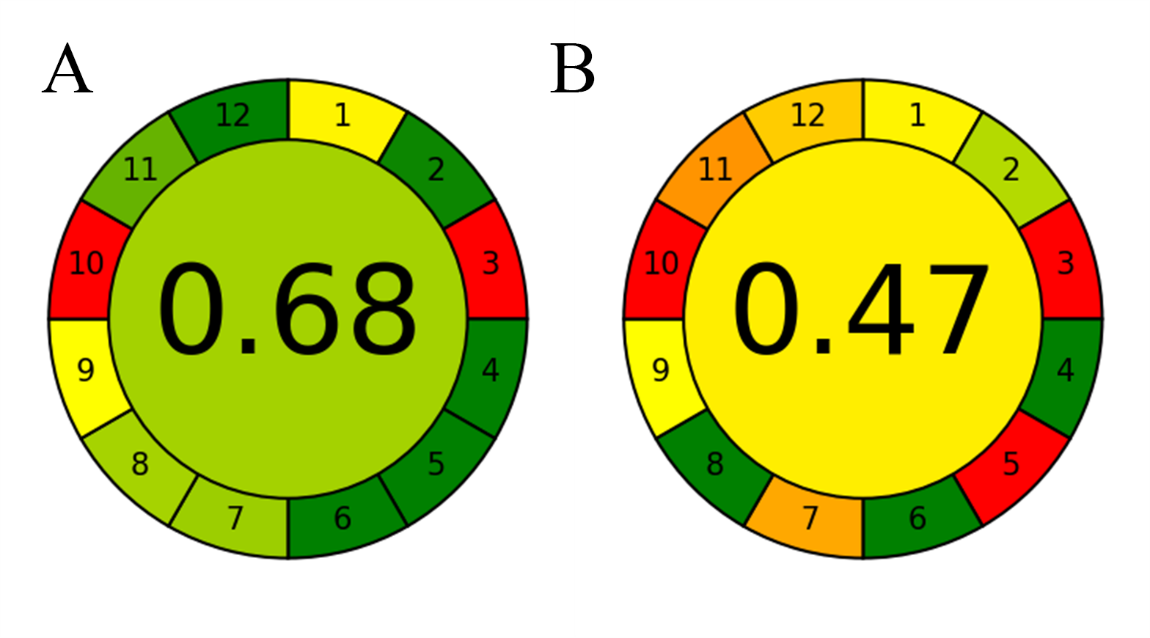


**Figure S-7 The software "AGREE" evaluates this method and the standard method on "green".** A, B refer to this method and standard method, respectively.
