# Supplementary figures and images for: Rapid and sensitive determination of Se and heavy metals in foods using electrothermal vaporization inductively coupled plasma mass spectrometry with a novel transportation system
Source: Front Nutr. 2023 Jun 7;10:1201801. doi: 10.3389/fnut.2023.1201801 (PMC10282127; doi:10.3389/fnut.2023.1201801)

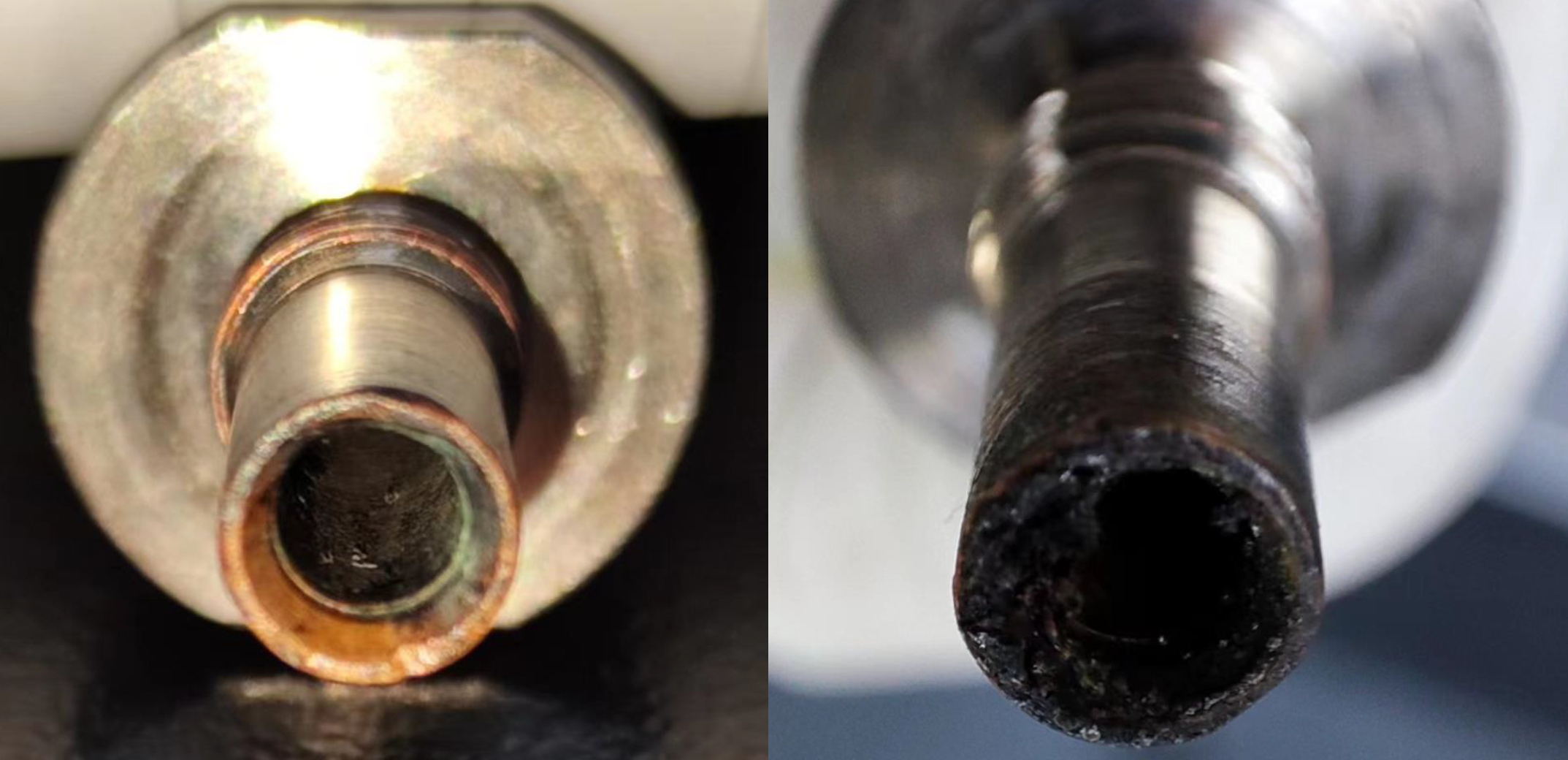

Supplement: Supplementary file 2 [file Image_1.TIF]

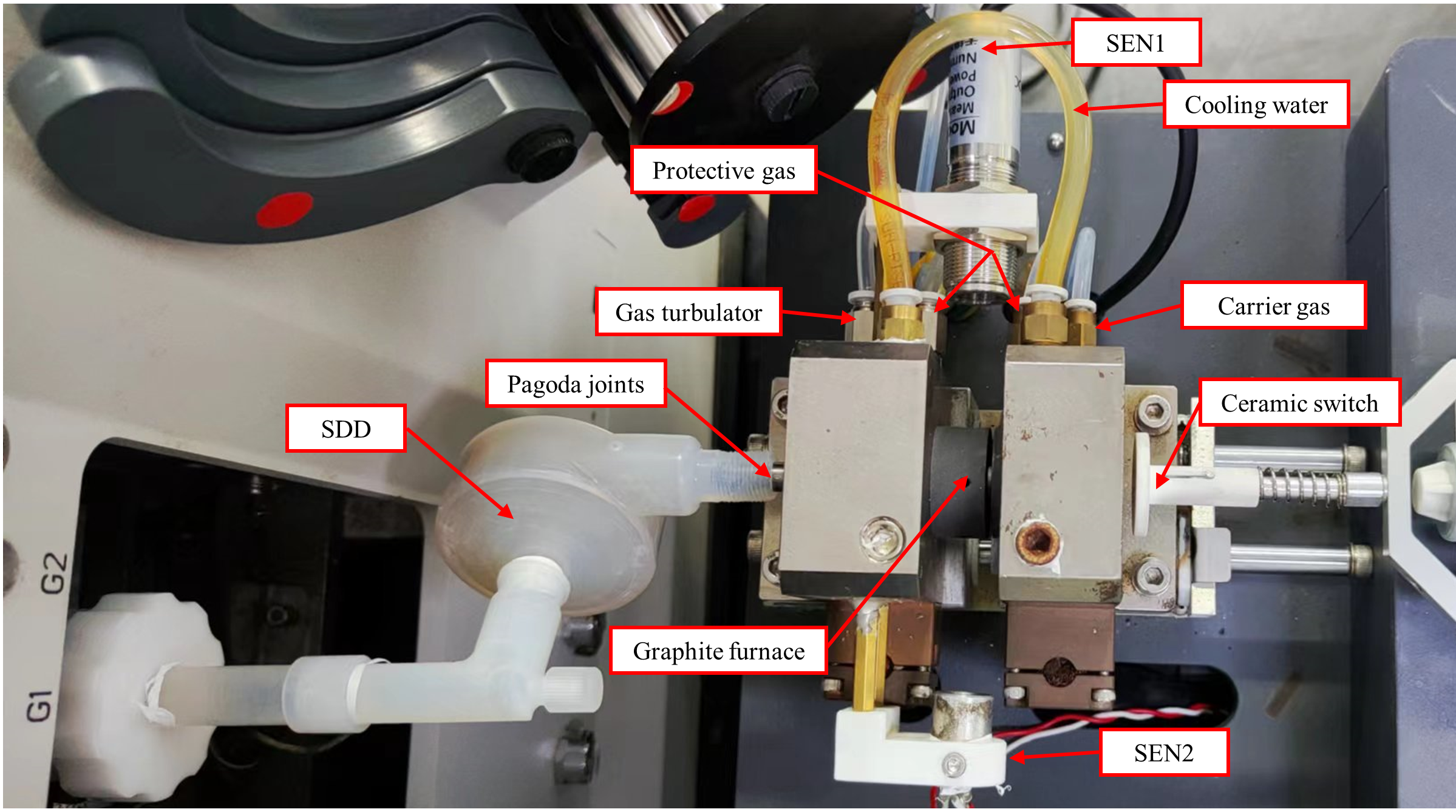

Supplement: Supplementary file 3 [file Image_2.TIF]

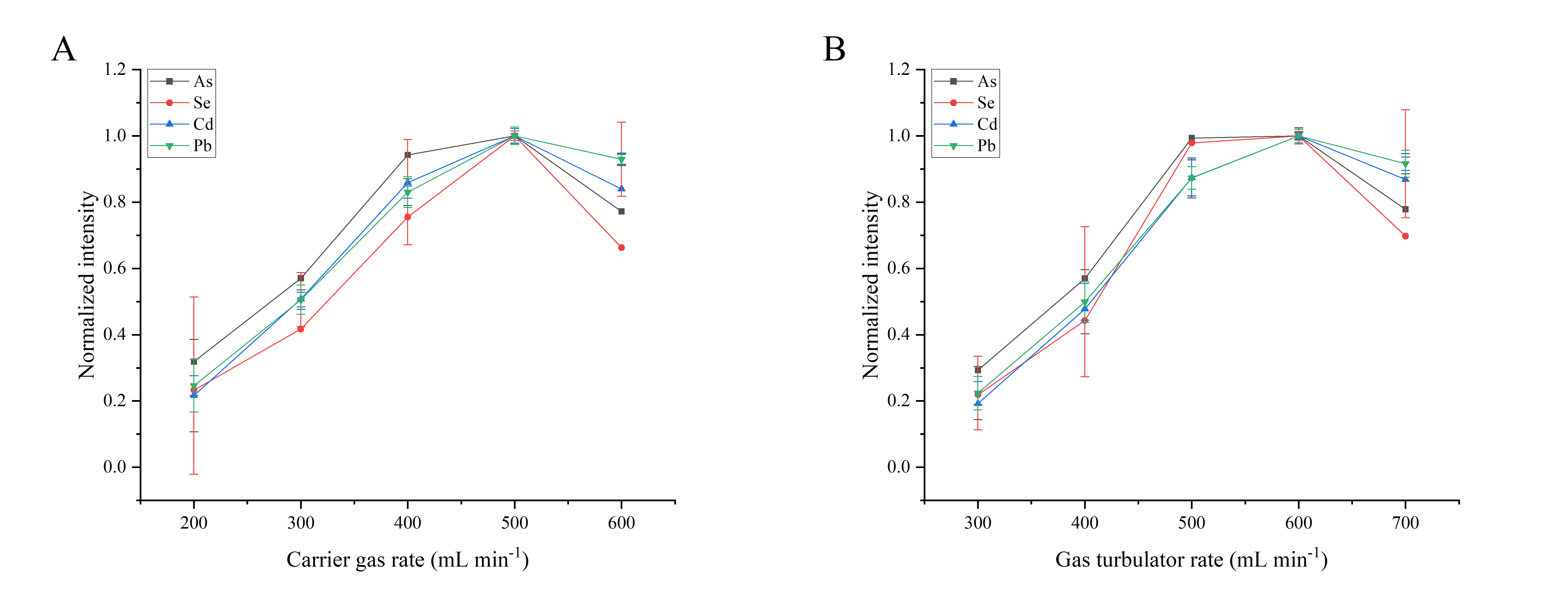

Supplement: Supplementary file 5 [file Image_4.TIF]

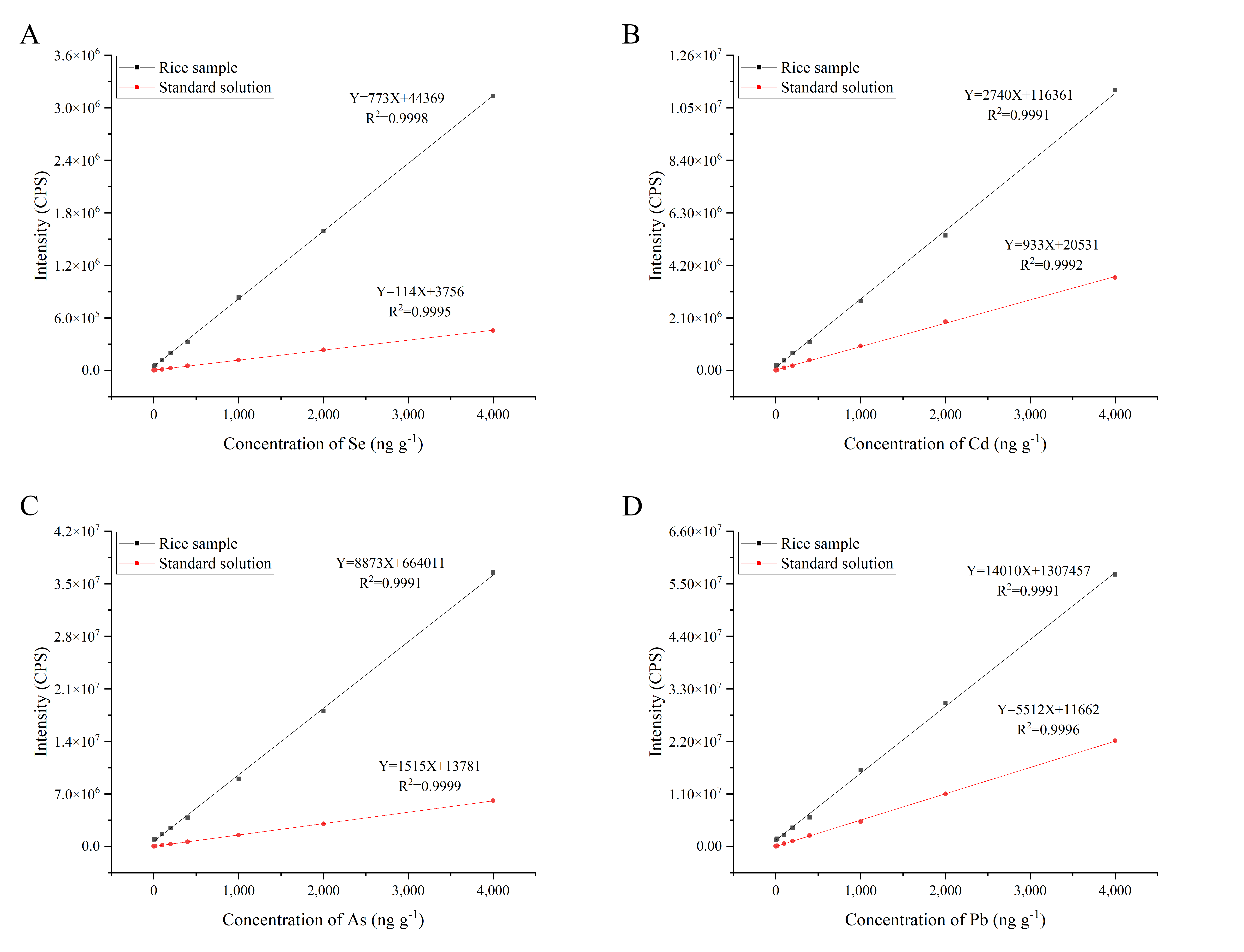

Supplement: Supplementary file 6 [file Image_5.TIF]
